# Supplementary material for: Probiotic Properties of Lactic Acid Bacteria with High Conjugated Linoleic Acid Converting Activity Isolated from Jeot-Gal, High-Salt Fermented Seafood
Source: Microorganisms. 2021 Oct 28;9(11):2247. doi: 10.3390/microorganisms9112247 (PMC8625341; doi:10.3390/microorganisms9112247)
Supplement: Supplementary file 1 [file microorganisms-09-02247-s001.zip › microorganisms-1363016-supplementary.pdf]

<u>Supplementary Materials</u>

Probiotic Properties of Lactic Acid Bacteria with High Conjugated Linoleic Acid Converting Activity Isolated from *Jeot-Gal*, High-Salt Fermented Seafood

Song et al.

<u>Contents</u>

Supplementary Table S1.
Primer sequences of cytokines used in qRT-PCR analysis.

Supplementary Figure S1.
MRS plate agar medium containing 0.2% linoleic acid. (a) large and well growth colony.

Supplementary Figure S2.
UV spectral scan of hexane extracts. (a) fatty acids extracted from the fermentation culture of JBCC105611, (b) fatty acids extracted from the fermentation culture of JBCC105657.

Supplementary Figure S3.
Phylogenetic tree based on 16S rDNA analysis of LAB isolated from *Jeot-gal*s.

Supplementary Figure S4.
Antibacterial activity of some CLA-producing LAB against pathogenic bacteria. (a) *Stapylococcus aureus* KCTC 1916, (b) *Staphylococcus epidermidis* KCTC 1917, (c) *Staphylococcus xylosus* KACC 13239, (d) *Pseudomonas aeruginosa* KACC 10186, (e) *P. putida* KACC 10266, (f) *Bacillus cereus* KACC 10097, (g) *Bacillus subtilis subsp. spizizenii* KACC 14741, (h) *Bacillus vallismortis* KACC 12149, (i) *Escherichia coli* KACC 13821, (j) *Propionibacteria acnes* KCTC 3314.

Supplementary Figure S5.

The effects of selected *lactobacillus* strains on RAW 264.7 cell proliferation by using the MTT colorimetric assay.

Supplemental Table S1

Supplementary Table S1. Primer sequences of cytokines used in qRT-PCR analysis.

Gene		Primer sequences	Tm
IL-1β	Forward	5'-TGACGGACCCCAAAAGAT-3'	52.1
	Reverse	5'-GTGATACTGCCTGCCTGAAG-3'	52
IL-6	Forward	5′-CCGGAGAGGAGACTTCACAGAG-3′	56.6
	Reverse	5′-TCATTTCCACGATTTCCCAGAG-3′	57.5
TNF-α	Forward	5′-AGGCACTCCCCCAAAAGATG-3′	57.4
	Reverse	5′-CACCCCGAAGTTCAGTAGACAGA-3′	56.9
IL-10	Forward	5′-GCTGGACAACATACTGCTAACCGACTC-3′	62
	Reverse	5′-TCCTTGATTTCTGGGCCATGCTTCTCT-3′	66.4
IL-12	Forward	5′-CGTGCTCATGGCTGGTGCAAAG-3′	64.4
	Reverse	5′-CTTCATCTGCAAGTTCTTGGGC-3′	57.5
TGF-β	Forward	5′-GCTACCATGCCAACTTCTGTCTG-3′	57.4
	Reverse	5′-GAAGCGCCCGGGTTGTGTTGGTTGTAG-3′	70.7
GAPDH*	Forward	5'-CATGGCCTTCCGTGTTCCTAC-3'	57.9
	Reverse	5'-TCAGTGGGCCCTCAGATGC-3'	58

*GAPDH, Glyceraldehyde-3-phosphate dehydrogenase.

Supplemental Figure S1

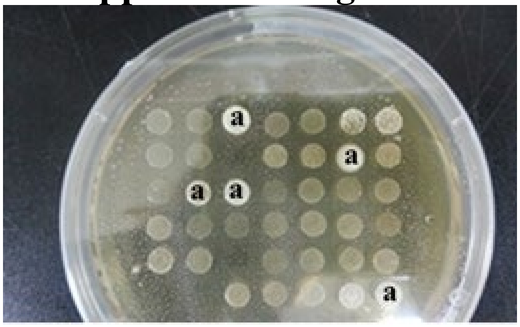

Supplementary Figure S1. MRS plate agar medium containing 0.2% linoleic acid (a) large and well colony.

Supplemental Figure S2

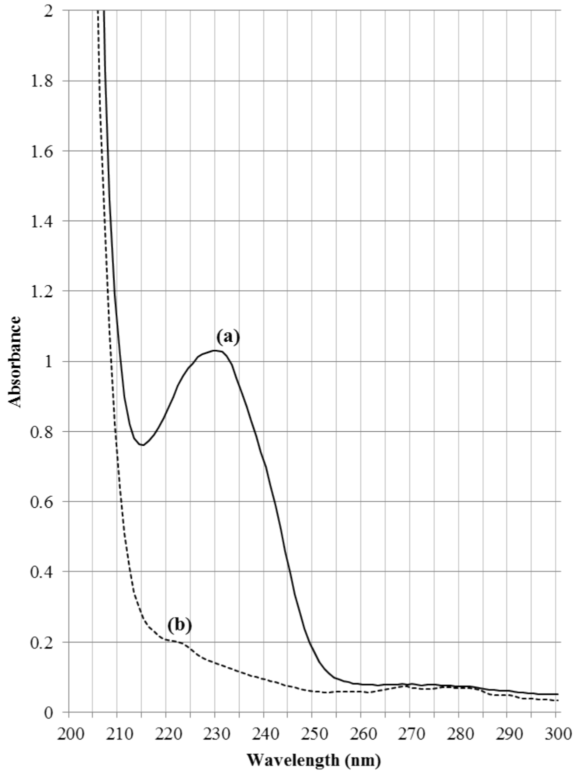

Supplementary Figure S2. UV spectral scan of hexane extracts. (a) fatty acids extracted from the fermentation culture of JBCC105611, (b) fatty acids extracted from the fermentation culture of JBCC105657.

Supplemental Figure S3

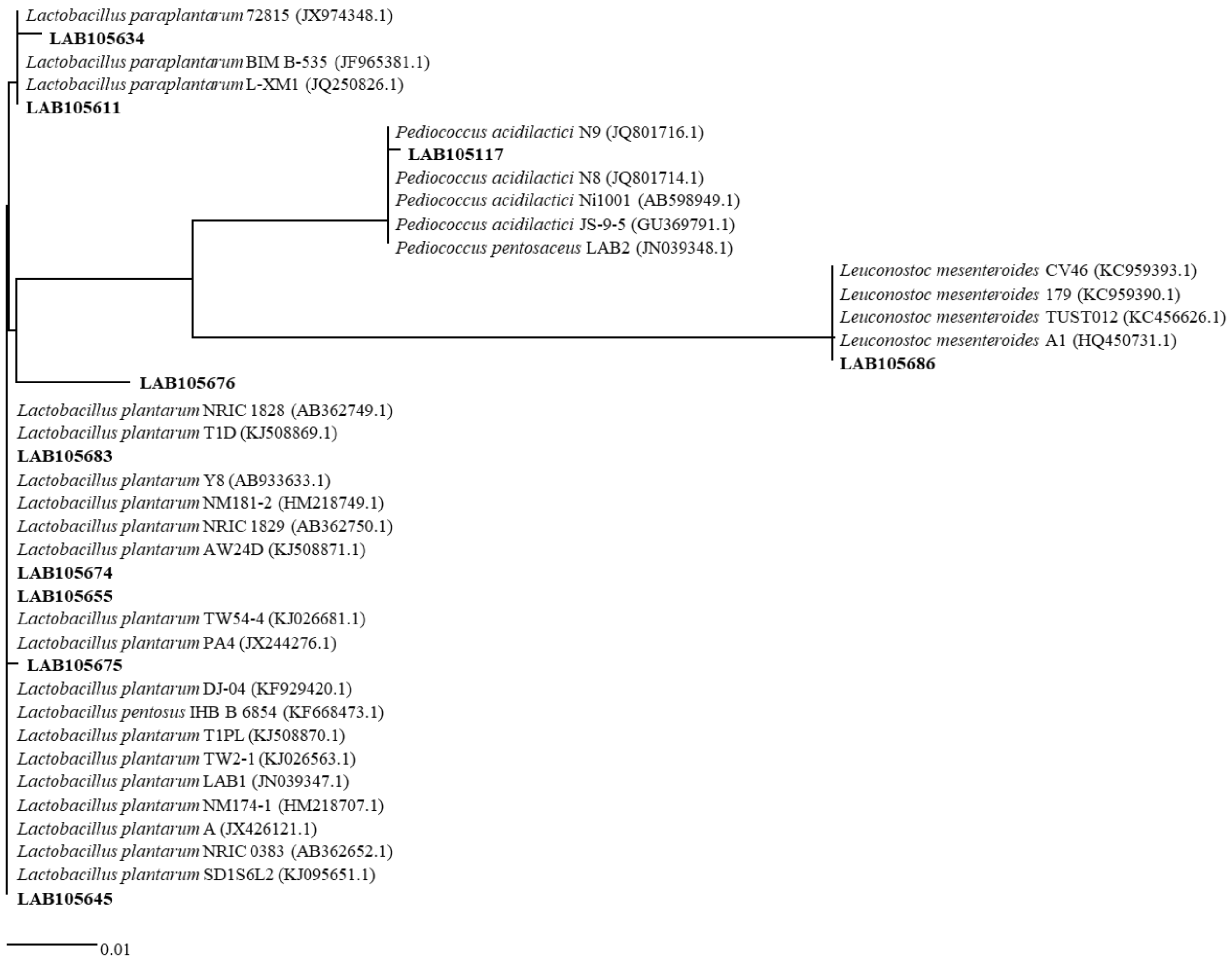

Supplementary Figure S3. Phylogenetic tree based on 16S rDNA analysis of LAB isolated from *Jeot-gal*s.

Supplemental Figure S4

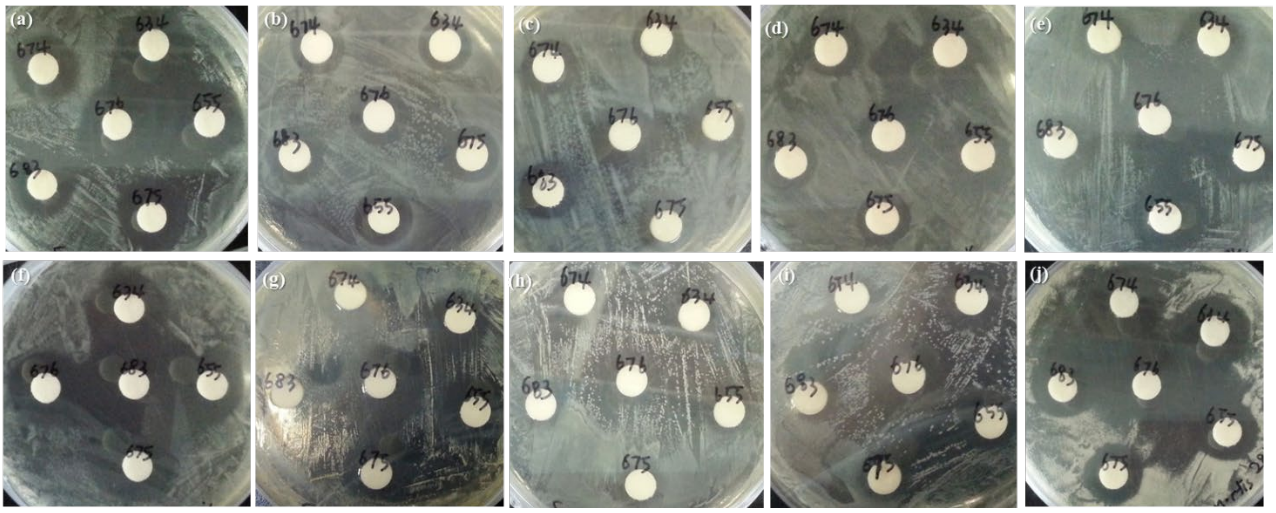

Supplementary Figure S4.

Antibacterial activity of some CLA-producing LAB against pathogenic bacteria. (a) *Stapylococcus aureus* KCTC 1916, (b) *Staphylococcus epidermidis* KCTC 1917, (c) *Staphylococcus xylosus* KACC 13239, (d) *Pseudomonas aeruginosa* KACC 10186, (e) *P. putida* KACC 10266, (f) *Bacillus cereus* KACC 10097, (g) *Bacillus subtilis subsp. spizizenii* KACC 14741, (h) *Bacillus vallismortis* KACC 12149, (i) *Escherichia coli* KACC 13821, (j) *Propionibacteria acnes* KCTC 3314.

Supplemental Figure S5

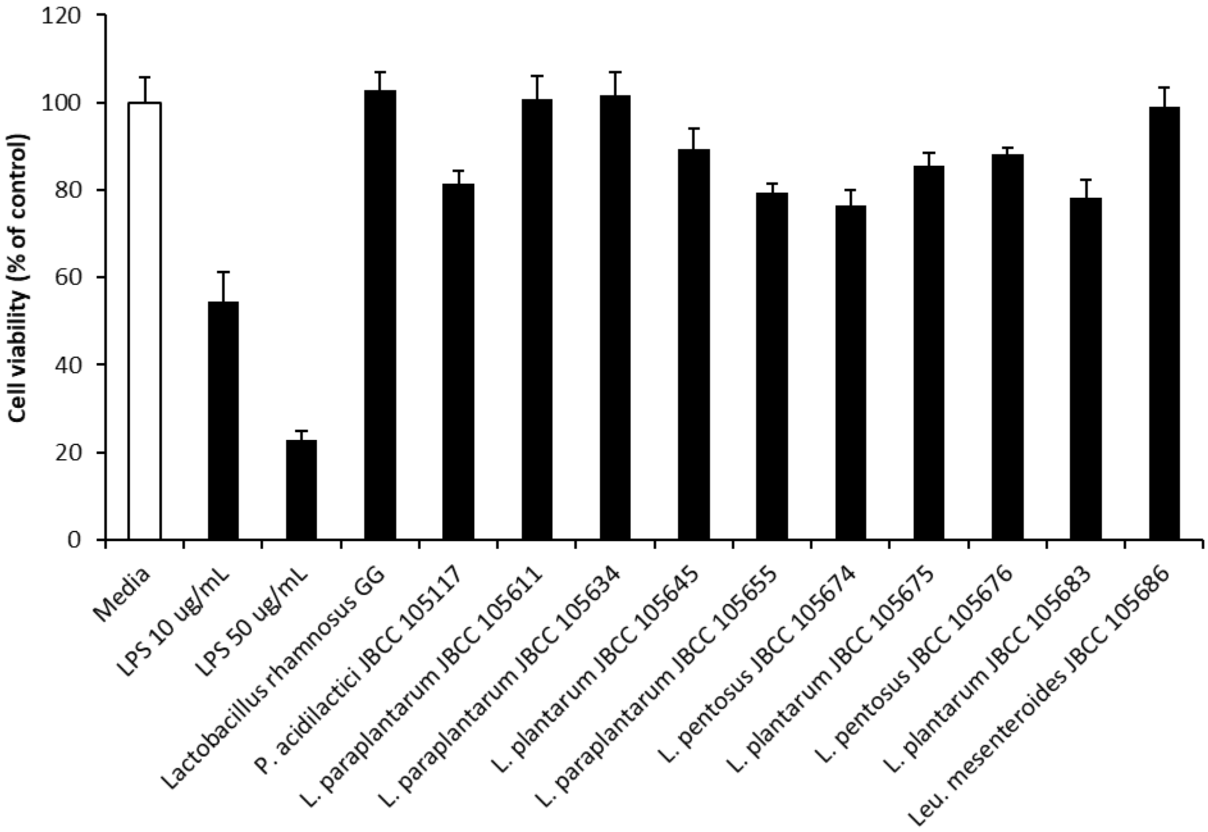

Supplementary Figure S5. The effects of selected *lactobacillus* strains on RAW 264.7 cell proliferation by using the MTT colorimetric assay.
